# Supplementary material for: Anti-Obesity Effects of Multi-Strain Probiotics in Mice with High-Carbohydrate Diet-Induced Obesity and the Underlying Molecular Mechanisms
Source: Nutrients. 2022 Dec 5;14(23):5173. doi: 10.3390/nu14235173 (PMC9739441; doi:10.3390/nu14235173)
Supplement: Supplementary file 1 [file nutrients-14-05173-s001.zip › nutrients-2063743-supplementary.pdf]

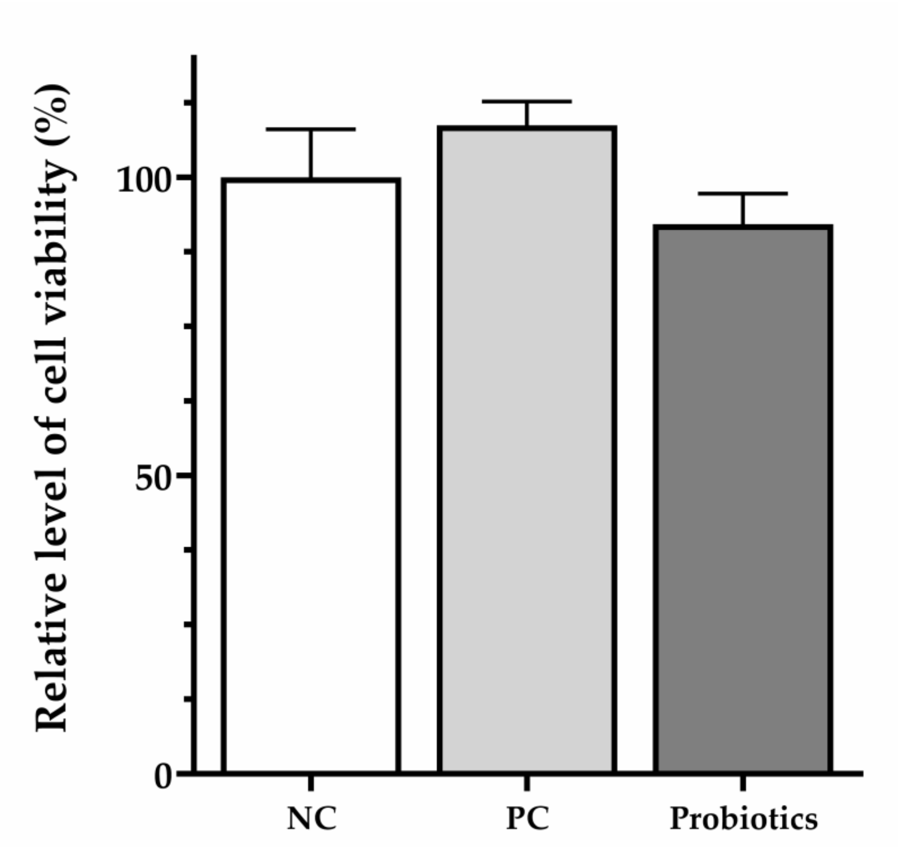

Supplementary Figure S1. Cell viability (MTT assay). Legend: NC, negative control; PC, orlis tat-treated cells.

Supplementary Table S1. Serum concentrations of hormones associated with the developmen t of obesity

	Leptin (μg/ml)	Adiponectin (μg/ml)	Insulin (μg/ml)
ND	6.92 ± 0.94	12.45 ± 1.21	0.93 ± 0.07
HCD + Placebo	65.13 ± 5.07 ***	6.34 ± 0.82 ***	1.77 ± 0.10 ***
HCD + Probiotics	45.01 ± 3.02###	8.10 ± 0.86###	1.34± 0.08###

Data are expressed as mean ± standard deviation (n=5 per group). *** p<0.001 vs ND; ### p<0.001 vs HCD + placebo. Legend: HCD, high carbohydrate diet; ND, normal diet.
